# Feasibility of Detecting Natural Frequencies of Hydraulic Turbines While in Operation, Using Strain Gauges

**DOI:** 10.3390/s18010174

**Published:** 2018-01-10

**Authors:** David Valentín, Alexandre Presas, Matias Bossio, Mònica Egusquiza, Eduard Egusquiza, Carme Valero

**Affiliations:** Center for Industrial Diagnostics and Fluid Dynamics (CDIF), Polytechnic University of Catalonia (UPC), Av. Diagonal, 647, ETSEIB, 08028 Barcelona, Spain; alexandre.presas@upc.edu (A.P.); matias.alberto.bossio@upc.edu (M.B.); monica.egusquiza@upc.edu (M.E.); eduard.egusquiza@upc.edu (E.E.); m.del.carmen.valero@upc.edu (C.V.)

**Keywords:** natural frequencies, hydraulic turbine, strain gauges, modal analysis

## Abstract

Nowadays, hydropower plays an essential role in the energy market. Due to their fast response and regulation capacity, hydraulic turbines operate at off-design conditions with a high number of starts and stops. In this situation, dynamic loads and stresses over the structure are high, registering some failures over time, especially in the runner. Therefore, it is important to know the dynamic response of the runner while in operation, i.e., the natural frequencies, damping and mode shapes, in order to avoid resonance and fatigue problems. Detecting the natural frequencies of hydraulic turbine runners while in operation is challenging, because they are inaccessible structures strongly affected by their confinement in water. Strain gauges are used to measure the stresses of hydraulic turbine runners in operation during commissioning. However, in this paper, the feasibility of using them to detect the natural frequencies of hydraulic turbines runners while in operation is studied. For this purpose, a large Francis turbine runner (444 MW) was instrumented with several strain gauges at different positions. First, a complete experimental strain modal testing (SMT) of the runner in air was performed using the strain gauges and accelerometers. Then, the natural frequencies of the runner were estimated during operation by means of analyzing accurately transient events or rough operating conditions.

## 1. Introduction

Hydropower is one of the main electricity generation sources in the world. About 13% of the electricity generated in 2015 was provided by hydropower [1]. Moreover, with the entrance in the energy market of new renewable energies in the last 10 years, such as wind or solar power, hydropower is playing an essential role due to its fast response and regulation capacity. Wind and solar power strongly depend on the weather conditions, so their generation is not constant. This requires more regulation and a fast response from other electricity-generation sources, which can only be provided by hydropower. Hydropower is able to regulate its power in a wide range (from 20% to 100% of maximum power) with a fast response (start-up, stop and changes in loads in less than one minute). Moreover, it can store large amounts of energy by using reversible pump-turbine (RPT) power plants when there is a surplus of energy.

Under these circumstances, hydraulic turbines are increasingly working in off-design conditions, far from their best efficiency point (BEP). Moreover, they are subjected to transient events (start/stop and load changes) many more times in a day than some years ago. In terms of the lifetime of the hydraulic turbine components, this is worse than working at the BEP, especially for the runner [2]. The runner is the rotating element that transforms the energy of the water into mechanical energy, so it is the most critical part of the machine in terms of mechanical stresses. Some failures in hydraulic turbine runners have been documented in the last few years due to fatigue or mechanical resonance [3,4,5,6]. Therefore, the dynamic response of the runner has to be well-known in order to avoid these fatigue or resonance problems. This means that it is of paramount importance to determine accurately the natural frequencies, damping ratios and mode-shapes of the runner in operation.

To determine experimentally the dynamic response of hydraulic turbine runners in operation is challenging, especially in the case of Francis turbines and pump turbines, because the runner of those types of machines is rotating and confined in water, with small gaps and seals, and is strongly affected by boundary conditions [7,8,9]. The natural frequencies of this kind of hydraulic turbine runner are affected by the added mass of the surrounding water [10,11], the rotation [12,13] and the distances to the stationary parts [7,14]. Numerical models are commonly used to estimate these natural frequencies [2,10,14] but they present some uncertainties due to the complexity of the problem, and usually experimental results are needed to calibrate them.

To estimate the mechanical stress and strain of the runner for different operating conditions, strain gauges have been used in the past [2,15,16]. These studies confirmed that the worst operating conditions in terms of mechanical stress are the start-ups, stops and low loads (power less than 50% of maximum power). However, they have never been used to determine the natural frequencies of the runner in operation. The method of determining the dynamic response of a structure using strain gauges is called strain modal testing (SMT) [17,18]. Mucchi [18] compared this method with the conventional method used to determine the dynamic response of structures by means of displacement, velocity or acceleration sensors. He presented the difficulties and drawbacks of using strain gauges for this purpose, as well as a new piezoelectric strain sensor to perform SMT. Nevertheless, SMT was applied only to a simple structure as in the case of a cantilever plate, but has been never applied in a complex structure such a hydraulic turbine runner.

In this paper, as a first step, a SMT is performed in a large Francis turbine runner (444 MW of rated power) in air, and the results are compared with a conventional experimental modal analysis (EMA) using accelerometers. To do so, the runner was impacted in different positions and the response was measured with several strain gauges and accelerometers. In this way, the natural frequencies and mode shapes of the runner were identified and classified using both kinds of sensors. The next step was to identify the natural frequencies and mode shapes of the runner while in operation. For this purpose, the response measured with the strain gauges during several transient events, such as start-ups and stops, as well as during rough operating conditions, has been analyzed in detail. Wavelet time–frequency methods and frequency-spectrum averaged methods were applied in the analysis of the strain-gauge signals in order to estimate the natural frequencies of the runner in operation.

## 2. Dynamics of a Francis Turbine Runner

In this section, the dynamic behavior of Francis turbine runners is discussed. To study the dynamic behavior of a Francis turbine runner, both dynamic response and excitation of the runner need to be defined. A general overview of the dynamic response of Francis turbines runners is presented in Section 2.1, and all possible sources of excitation in Francis turbines are discussed in Section 2.2.

### 2.1. Modal Behavior of a Francis Turbine Runner

The mode shapes of all kinds of Francis turbine runners are similar, especially those with a similar specific speed value, since they have common geometrical characteristics [5,19,20]. These mode shapes are formed by different number of nodal diameters (ND) with different relative amplitude between the crown, band and blades [8,21]. If the deformation in the crown is bigger than in the band or in the blades, these mode shapes are called crown-dominant mode shapes (CD) [9]. If the deformation in the band is the greatest, these mode shapes are called band-dominant mode shapes (BD); and if it is in the blades, they are called blade-dominant modes (BlD) [21]. In the case that the deformation is almost the same in the different parts of the runner, the mode shapes are called global mode shapes (G) [21]. Normally, the first natural frequencies that appear are global mode shapes (G) and, then, the natural frequencies appear grouped according to the mode shape type (CD, BD or BlD).

The mode-shapes are generally not modified when submerging the runner in water, but the natural frequencies decrease in comparison to the situation in air [10,22,23,24] due to the added mass effect. Moreover, this added mass effect is stronger for nearby rigid surfaces [8]. The natural frequencies can decrease about 50% from their value in air when the runner is submerged and confined in water, but this value is totally dependent on the mode shape. Some studies [10,22,23,24] have confirmed that the higher the nodal diameter (ND) of the mode shape, the higher the affect on the natural frequency of the runner in water in comparison with that in air. Furthermore, the added mass effect is also stronger for the mode shapes with relative displacement between crown, band or blades (CD, BD or BlD mode shapes) than for the global mode shapes (G) [11]. Other parameters such as the temperature and density of the surrounding fluid can also affect the natural frequencies of the runner. The temperature directly affects the stiffness of the structure: when increasing temperature, stiffness decreases and, therefore, the natural frequencies also decrease [25]. The density of the surrounding fluid is an important parameter in the added mass effect: the higher the density of the surrounding fluid, the smaller the natural frequencies of the submerged structure [7,26]. Nevertheless, in the hydraulic turbomachinery field, temperature and density changes are usually small.

The order of appearance of the mode shapes in frequency may be different in water than in air because the affect of the water is different for every mode shape. Hence, mode shapes need to be identified when the runner is submerged in water, in mounting conditions, and in operation.

### 2.2. Excitation Phenomena in Francis Turbines

During operation, Francis turbines are subjected to different hydraulic excitation phenomena. These excitation phenomena may be periodic, transient or random [27] depending on their origin. Due to the nature of these excitation phenomena, the natural frequencies of the runner may be excited and detected. Therefore, every excitation phenomena should be studied in detail to know if the runner natural frequencies can be excited by the hydraulic phenomenon or not, and if it is the case, which can be excited and which cannot.

#### 2.2.1. Periodic Excitation

In this section, the main kinds of periodic excitation sources in Francis turbines are explained and their possibility for exciting runner natural frequencies is discussed.

• Rotor-Stator Interaction

The most important periodic excitation in a Francis turbine is given by the rotor-stator interaction (RSI) [28,29,30]. The pressure field at the inlet of the runner is affected by the interaction of the runner blades and the guide vanes. When the rotating blades of the runner pass in front of the static guide vanes, the pressure field in the gap between blades and vanes can be described as the superposition of all the combinations m, n [28,29]:

(1) $$P_{m,n}\left(\theta ,t\right)=A_{m,n}\cdot \cos\left({\mathrm{mZ}}_{v}\theta_{s}+\Psi_{m}\right)\cdot \cos\left({\mathrm{nZ}}_{b}\theta_{r}+\Psi_{n}\right),$$

In Equation (1), θ is the angular coordinate (index s is for the stationary coordinate and r for the rotating coordinate); Z_v_ is the number of guide vanes and Z_b_ the number of runner blades; and m, n are integer numbers (1, 2, …, ∞) that represent the order of harmonic. The amplitude of the excitation A_m,n_ depends on several parameters like the head (difference in altitude between the upper and the lower reservoir), operating point, design of the machine and order of harmonics (m, n). Therefore, RSI excitation is a sum of sinusoidal waves at different frequencies with different amplitudes. From the rotating frame or from the runner point of view, these frequencies are calculated as in Equation (2), where f_f_ is the runner rotating frequency. The shape of the wave (k_m,n_) associated with each f_m_ is a combination of the n harmonics of Z_b_ and the m harmonics of Z_v_ (Equation (3)).

(2) $$f_{m}=m\cdot Z_{v}\cdot f_{f},$$

(3) $$k_{m,n}=n\cdot Z_{b}-m\cdot Z_{v}$$

Thus, during operation in a steady condition, the runner is receiving an excitation due to the RSI at the frequencies f_m_ with the shape k_m,n_. For example, for a combination of Z_b_ = 16 runner blades and Z_v_ = 20 guide vanes and a rotating speed of f_f_ = 2.14 Hz (128.6 rpm), the main frequencies of excitation from the rotating frame are: f_1_ = 42.8 Hz, f_2_ = 85.6 Hz, f_3_ = 128.4 Hz and f_4_ = 171.2 Hz. The shape corresponding to each of those frequencies is given in Table 1, which is compiled according to Equation (2). The positive sign of the values in Table 1 means that the wave is rotating in the same direction as the runner, and the negative sign that it is rotating in the opposite direction. The shape of the excitation frequency f_1_ corresponds to the first column of Table 1. In this case, it is a sum of a wave with 4 nodal diameters rotating in the opposite direction to the runner and waves with 12, 28, 44, 60, … nodal diameters rotating in the same direction as the runner. Only the smaller values of k_m,n_ are usually considered because they are the ones that present higher amplitudes [31,32] (colored in red in Table 1). Therefore, f_1_ is mainly a wave with 4 nodal diameters rotating in the opposite direction to the runner. In that way, f_2_ is a −8, +8 wave, f_3_ is a +4 wave and f_4_ a 0 nodal diameters wave.

According to Presas et al. [33], resonance only occurs if the excitation frequency matches in value and in shape with the natural frequency and its mode-shape. Therefore, in steady conditions, where the rotating speed is constant in Francis turbines, the natural frequencies of the runner will only be excited by the RSI excitation if they match in value and in shape with the RSI excitation.

However, during a start-up or stop of the Francis turbine, the rotating speed (f_f_) increases from 0 to the nominal value in the case of the start-up and vice versa for the stop. This procedure of acceleration and deceleration of the runner can be of about 30 s of duration. This means that the excitation frequency of the RSI also changes from 0 to its steady value (f_m_) or vice versa (see Equation (2)). Therefore, if the natural frequency of the runner is below the steady value of the excitation frequency (f_m_) and the excitation shape coincides with the mode shape of the runner, a resonance is expected to happen. In this case, this natural frequency may be detected every start-up or stop. For instance, taking the values of the same example presented before (Z_b_ = 16, Z_v_ = 20 and f_f_ = 2.14 Hz), if the natural frequency of the runner associated to a 4ND mode-shape is below f_1_ = 42.8 Hz, this will be excited during the start-up and stop of the machine. The same applies for the rest of the excitation frequencies (f_2_, f_3_, f_4_, …, f_∞_) and the rest of the natural frequencies of the runner.

• Draft Tube Cavitation Surge

In Francis turbines at part load (50–80% BEP flow rate) and overload (above BEP flow rate), a periodic hydraulic phenomenon related to cavitation appears in the runner outlet and draft tube. This phenomenon is called the vortex rope and it has been studied for many years [34,35,36]. It is a core formed by a variable volume of vapor rotating in the same direction as the runner for the part-load operation, and in the opposite direction to the runner for the overload operation. For the part-load vortex rope, this takes a helical shape and presents a precession rotation at 0.25–0.35 times the runner rotating speed (f_f_). In this case, circumferential pressure pulsations are generated at this low frequency, displacing the runner in the radial direction. For the overload vortex rope, the vortex is axially centered in the draft tube cone, inducing an axial force over the runner. The frequency of this overload vortex rope is in the same range as the part-load vortex rope. As this phenomenon occurs at a very low frequency, normally it does not match any natural frequency of the runner.

However, strong pressure fluctuations may occur if the vortex rope frequency matches one of the free natural-oscillation frequencies of the draft tube, penstock or the whole hydraulic circuit. This provokes large bursts of pressure pulses in the draft tube, causing strong vibrations on the turbine and even on the power-house. These phenomena are called part-load resonance or overload resonance [36,37]. During them, the runner receives large pressure pulses, which can act as strong impacts over the structure, exciting all the natural frequencies of the runner. Therefore, this phenomenon becomes a transient excitation source at the same time as it is periodic (0.25–0.3 f_f_).

#### 2.2.2. Transient Excitation

One can understand transient excitations as those that excite the structure during very short periods of time. This is the case of an impact made with an instrumented hammer in an EMA [38]. This kind of excitation is a broadband-type of excitation. In Francis turbines, this type of excitation is found at the very beginning of every transient event (start-up, stop, change in loads…). When the guide vanes start to turn, the flow rate passing through the runner changes, and this may act as an impact over the runner, exciting all its natural frequencies. The faster this process, the stronger the impact on the runner [39]. This phenomenon was previously analyzed in hydraulic turbines by Aguila et al. [40] to detect the natural frequencies of the rotor.

Furthermore, as mentioned in the section above, during part-load or overload resonances, the runner receives strong pressure fluctuations which also act as strong impacts over the runner, exciting its natural frequencies.

These phenomena occur in a very short time, and therefore the detection of the natural frequencies of the runner is challenging and joint time–frequency methods with high resolution in time and in frequency need to be applied in signal processing.

#### 2.2.3. Random Excitation

When Francis turbines work at very low loads (DPL, deep part load), a random hydraulic phenomenon related to the very turbulent flow appears [41]. The nature of this phenomenon is completely stochastic and it is very difficult to estimate. Therefore, during the operation at this point, the runner is excited with a random broadband excitation that is able to excite the different natural frequencies of the runner. This phenomenon is even more important when the machine is working at off-design heads [6]. Moreover, similar behavior happens when the machine is at speed no load (SNL), where the flow rate is very small and the machine turns but without being synchronized with the electrical grid [42]. This happens for a short time after every start-up.

Another random source of excitation that appears in Francis turbines is the cavitation, especially the leading edge cavitation or the travelling-bubble cavitation [34]. The first appears for higher heads than the design head in the inlet of the runner and the second is dependent on the lower reservoir level and appears at the blades’ outlet. In both cases, the cavitation bubbles collapse randomly, provoking erosion in the runner. These are very noisy types of cavitation, so they can excite the runner in a wide range of frequency. Moreover, it should be considered that the cavitation phenomena can affect the natural frequencies of the runner, since the added mass and stiffness of the water changes with the appearance of vapor cavities [43].

In this case, it is also difficult to detect the natural frequencies of the runner, since the excitation is always an unknown, so joint time–frequency signal-processing methods or spectrum-averaged methods are needed.

## 3. Experimental Tests

### 3.1. Description of the Selected Turbine

For this study, a large medium-head Francis turbine was selected. This Francis Turbine has a specific speed (n_s_) of 46 (further information about hydraulic turbines’ specific speed can be found in [20]) and a rated power of 444 MW. The study of the dynamic behavior of this Francis Turbine is part of the collaborative European Project Hyperbole (FP7-ENERGY-2013-1) [44]. The runner has 16 blades (Z_b_ = 16), whereas the distributor has 20 guide vanes (Z_v_ = 20). The rotating speed of the machine is 128.6 rpm (2.14 Hz). Taking advantage of an overhaul in the power plant, the machine was accessible enough to install several sensors in the rotating parts and in the stationary parts. The objective of these tests was to identify all the phenomena that occur in every operating point and how they are detected with the different sensors.

### 3.2. Instrumentation

**Figure 1:**
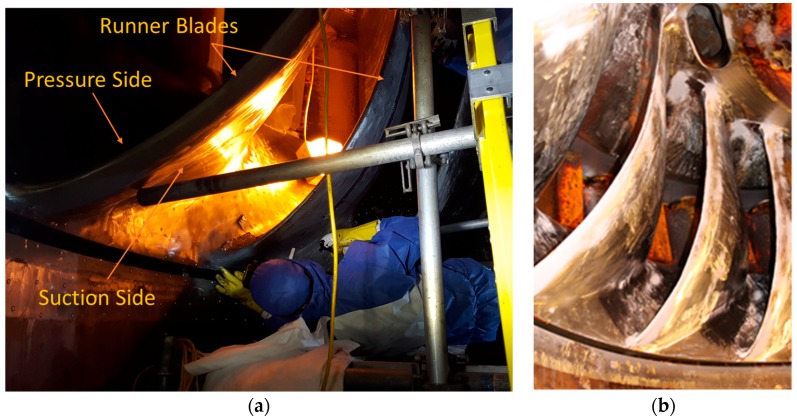
Pictures of the Francis turbine runner studied. (**a**) Picture during the installation of the strain gauges in the runner; (**b**) detail of the runner blades.

**Figure 2:**
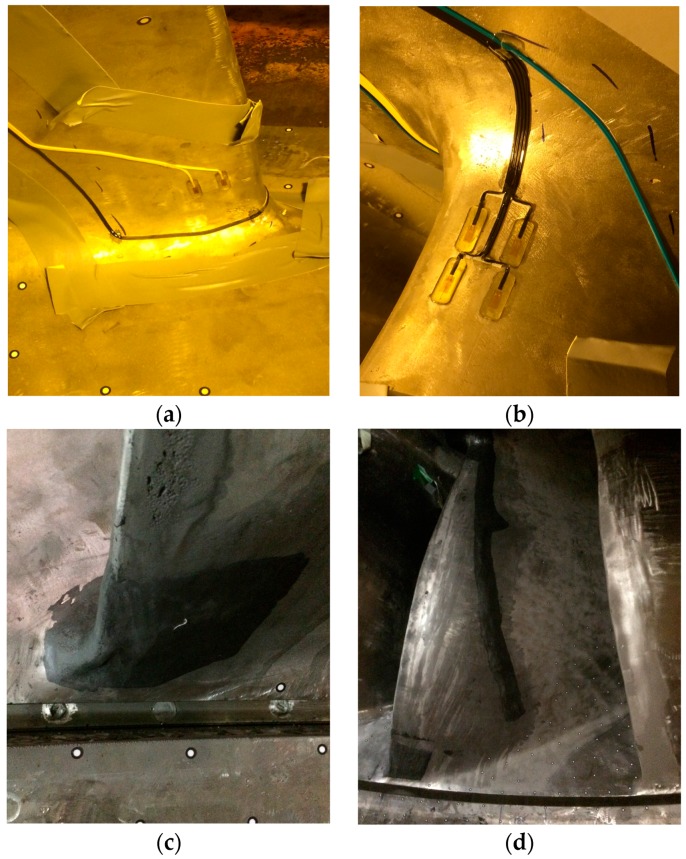
Detail of the strain gauges installed in the runner. (**a**) Strain gauges in the pressure side and band side; (**b**) strain gauges in the suction side and crown side; (**c**,**d**) detail of the epoxy resin covering the strain gauges.

The runner was instrumented with 24 strain gauges distributed in two different blades of the runner, separated by 180 degrees between them (blades numbers 7 and 15) (see Figure 1). Six strain gauges were installed in the pressure side of each blade, four in the crown side and two in the band side, and another six strain gauges were installed in the suction side of the blade, again four in the crown and two in the band side (see Figure 2). The strain gauges used had a gauge factor of 2, and a sensitivity of 3.266 mV/(μm/m). After gluing the strain gauges in the runner, they were covered with a special epoxy resin (Devcon Epoxy 14270) to protect them from the water. A detail of this resin applied to the gauges is seen in Figure 2c,d.

**Figure 3:**
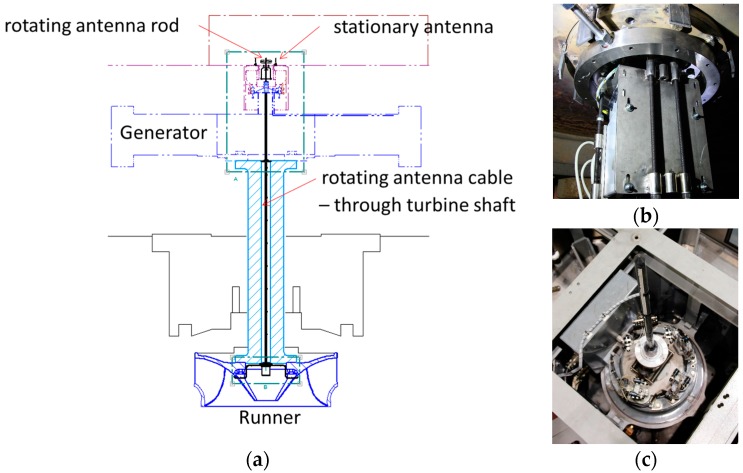
Detail of the telemetry system installed in the rotating train of the Francis turbine. (**a**) Sketch of the telemetry system; (**b**) picture of the telemetry system installed in the hub of the runner; (**c**) rotating antenna at the tip of the shaft.

The strain gauges were connected to a telemetry system located in the hub of the runner (Figure 3c) using a quarter of a Wheatstone bridge for each one. This telemetry system was connected to a rotating antenna located in the tip of the turbine shaft, in the generator side (Figure 3b). A stationary antenna was located near the rotating antenna to receive the signals of every strain gauge during operation. Then, this stationary antenna was connected to a signal conditioner and to an acquisition system (Bruel & Kjaer LAN-XI Type 3053). A sketch of this installation is seen in Figure 3. Once everything was connected, every strain gauge was calibrated onsite using the shunt calibration technique.

**Figure 4:**
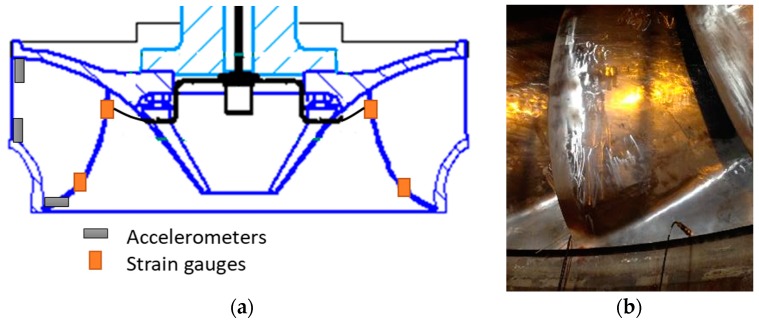
Detail of sensors available during the experimental modal analysis (EMA). (**a**) Sketch of the location of the accelerometers; (**b**) picture of the accelerometer located in the band outlet.

Aside from the strain gauges, three accelerometers were also provisionally installed in the runner to perform an EMA. The accelerometers (Kistler 8752A, 100 mV/g) were located axially at the inlet of the runner in the crown and in the band, and radially in the band outlet. A sketch of the accelerometers’ locations is shown in Figure 4. The EMA was carried out using an instrumented hammer Dytran 5802A (Dytran, Chatsworth, CA, USA) (sensitivity 220 μV/N).

### 3.3. Testing Procedure

First, an EMA was performed impacting the runner in 32 different points. During these tests, both strain gauges and accelerometers were installed. In that way, results obtained with strain gauges (SMT) could be compared with those obtained with the accelerometers. The runner was impacted radially in 16 positions of the band outlet (in the middle of every runner channel) and also radially in every blade (another 16 positions). For every point of impact, five different impacts were undertaken in order to compute the average of those impacts when analyzing. Accelerometers were not moved during the tests, only the impact position was changed. This method is called the roving hammer method.

Once the EMA and the SMT were finished, the machine was prepared for working again. This process took two days of isolation of the machine. The experimental tests while in operation consisted of testing the different operating conditions of the machine. The net head remained constant during the tests, which at that time was 1.06 times the rated net head for that machine. The operating conditions tested were the following:

1. Start-up (duration of 1 min): the machine goes from standstill to the nominal speed (128.6 rpm).
2. Speed no load (SNL): the machine turns at nominal speed but without generating energy (no excitation in the generator). Low periodic excitation due to RSI and important random excitation due to turbulence.
3. Deep part load (DPL): very low flow, so the machine was generating between 5% and 30% the rated power. Low periodic excitation due to RSI and important random excitation due to turbulence.
4. Part load (PL): between 30% and 70% of the rated power. Medium periodic excitation of the RSI and high periodic excitation due to part-load vortex rope.
5. Best efficiency point (BEP): for the head during the test, this point was at 90% of the rated power. High periodic excitation due to the RSI.
6. Overload (OL): Between 100% and 108% of the rated power. High periodic excitation due to the RSI and due to the overload vortex rope.

All the strain gauges worked properly during these tests.

## 4. Signal Processing

In this section, the signal-processing methods used for every analysis of the strain gauges signals are presented.

### 4.1. Strain Modal Testing

To analyze every point where the runner was impacted, a frequency response function (FRF) between the accelerometers or strain gauges and the hammer was computed for each one. The average in a frequency domain of 5 impacts was used taking 4 s of time signal and a frequency resolution of 0.25 Hz (1600 Hz maximum frequency analyzed). The coherence function was also computed between the accelerometers or strain gauges and the hammer in order to ensure the accuracy of the experimental testing. In that way, one FRF was obtained for every strain gauge (24 in total) and for every accelerometer (3 in total) for every point impacted (32 in total).

Using the 32 FRFs of one accelerometer, an operational deflection shape (ODS) of the runner can be obtained. Furthermore, natural frequencies and damping associated with every mode shape can be extracted using the complex mode indicator function (CMIF) method [38]. Once the mode shapes are known, the FRFs of the accelerometers and strain gauges can be compared.

### 4.2. Joint Time–Frequency Analysis

**Figure 5:**
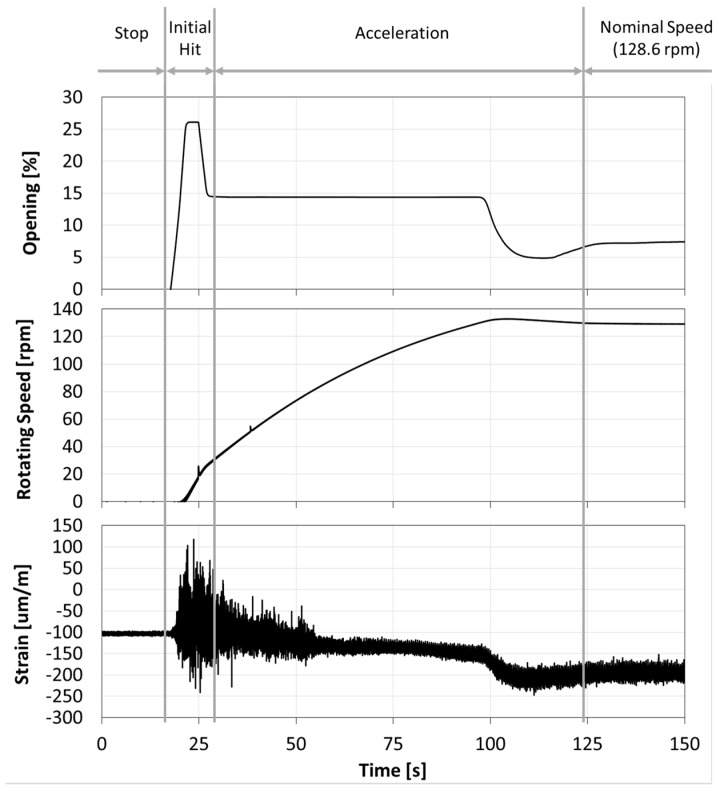
Time signal of the wicket gate opening, rotating speed and a strain gauge of the runner during the start-up of the machine.

To analyze the start-up and the stop of the machine, a joint time–frequency analysis with accurate resolution in frequency and in time is necessary. When the guide vanes start to open, the water hits the runner and excites its natural frequencies. Then, the machine starts to turn and increase its rotating speed. This process occurs in about 50 s and it is clearly seen in Figure 5. Moreover, to analyze the initial hit (see Figure 5), a joint time–frequency analysis is also needed. In both cases, a joint time–frequency analysis using Morlet wavelets has been used [45].

### 4.3. Averaged–Spectrum Analsysis

**Figure 6:**
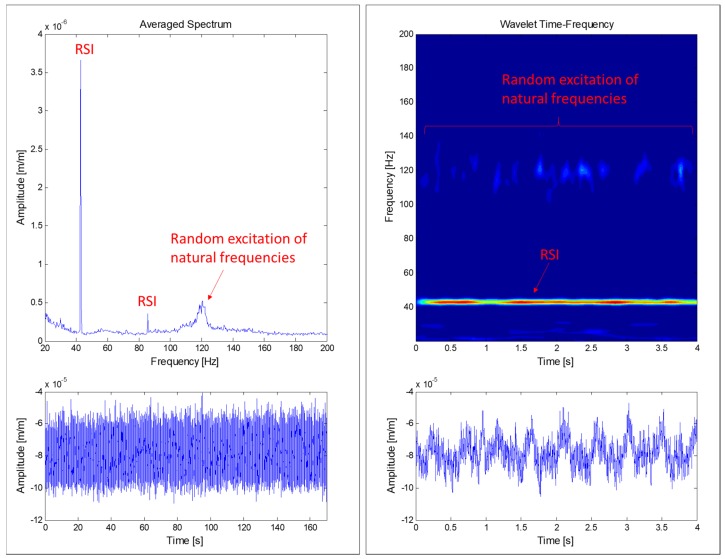
Comparison of an averaged-spectrum analysis (**left**) and a joint time–frequency analysis using wavelets (**right**) of a strain gauge signal during deep part load (DPL) operation.

The runner is subjected to a random excitation due to turbulence when the machine works at DPL. This kind of excitation excites the runner’s natural frequencies randomly, so a spectrum-average analysis is useful to detect them. A spectrum-average method is based on the average of several FFT (Fast Fourier Transform) along time. Some FFT may detect the runner’s natural frequencies and some FFT may not, since they are excited randomly along time. The average of all of the computed FFT permits us to estimate some of those excited natural frequencies. In this case, 100 averages during 170 s were used with a resolution of 0.25 Hz and maximum analyzed frequency of 400 Hz (see Figure 6). In comparison with a joint time–frequency method, this methods permits the analysis of more time signal with less computational resources. For a constant operating condition, this method emphasizes periodic and random excitations and vanishes transient short-time duration phenomena (see Figure 6).

## 5. Results

### 5.1. Natural Frequencies in Air

**Figure 7:**
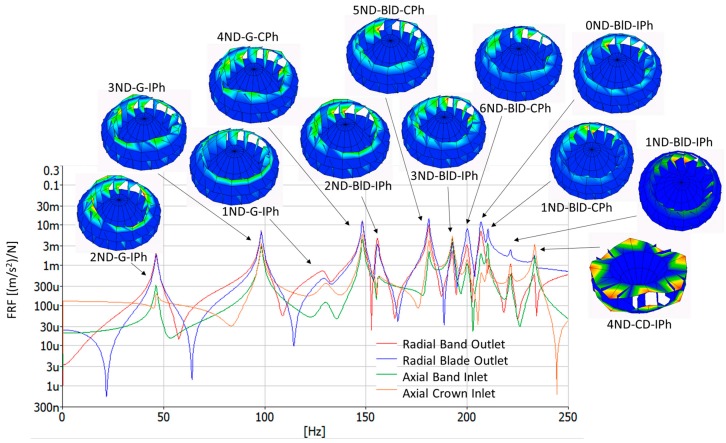
Frequency response function (FRF) in different points of the runner (0–250 Hz), as well as the operational deflection shape (ODS) of every runner mode shape associated with every natural frequency. Maximum displacement is colored red, and minimum displacement is colored blue. Videos of the mode shapes are found the Supplementary Materials section (S1 to S12).

The runner mode shapes, natural frequencies and damping ratios have been identified in air by means of the EMA and the ODS. They have been named according to the nomenclature explained in Section 2.1. Table 2 shows the first 12 mode-shapes, natural frequencies and damping ratios obtained within the frequency range 0–250 Hz with the EMA. Moreover, the ODS of these mode shapes are also seen in Figure 7 and in the supplementary videos S1 to S12 (see Supplementary Materials section). This picture also shows the FRFs of different points of the runner. It is clearly observed that there is a first zone (0 Hz to 150 Hz) where the band outlet deformation and the blades’ deformation is the same, so they have been called global modes (G). Then, there is another zone (150 Hz to 220 Hz) where the blade deformation is higher (BlD modes); whereas the last mode shape present higher deformation in the crown inlet than in the band outlet or the blades. This mode shape has been named the crown dominant mode shape (CD).

**Figure 8:**
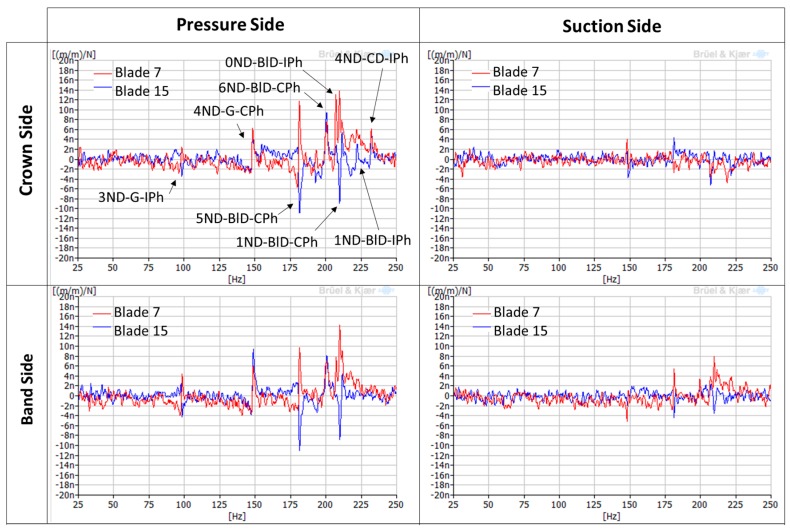
FRFs (only the real part) of different strain gauges with the hammer as a reference (impact in blade 7).

Once the mode shapes and natural frequencies have been identified with the EMA using accelerometers, it is necessary to see how these mode shapes are detected with all the different strain gauges installed in the runner. Figure 8 shows the FRFs of different strain gauges located in the runner when it was impacted in one of the blades, in this case, blade 7. They have been classified according to the side of the blade where they are located (pressure side or suction side) and if they are near the crown or the band (crown side or band side). It can be seen that, depending on the location of the strain gauge, natural frequencies are detected or not, and with different amplitudes. Only the real part of the FRF is shown in the picture to see graphically if the strain gauges of blade 7 and blade 15 (separated by 180°) are in phase or in counter-phase.

It can be observed that the natural frequencies are detected better on the pressure side of the blade. On the suction side of the blade, they are not detected or they present very low amplitudes. In comparison with the accelerometer, with the strain gauges only 8 natural frequencies are detected. Mode shapes 1, 3, 5, 7 (see Table 2) are not detected with the strain gauges, possibly because they present low local displacement in the location of the strain gauges or because the impact force was not enough to excite them. However, the rest of the mode shapes are detected with the corresponding phase between the two blades: 0ND, 2ND, 4ND and 6ND modes present in phase amplitudes in both blades, and 1ND, 3ND and 5ND modes present in counter-phase amplitudes in both blades. This information is essential for detecting every mode shape with the runner in operation.

### 5.2. Natural Frequencies under Operating Conditions

According to previous studies [10,22,23,24], the natural frequencies of Francis turbine runners decrease when they are confined in water. Depending on the mode shape and the confining distances, they can be of about 50% the natural frequency in air. In this section, the detection of the natural frequencies of the Francis turbine runner studied is discussed for different operating conditions of the machine.

#### 5.2.1. Start-Up

**Figure 9:**
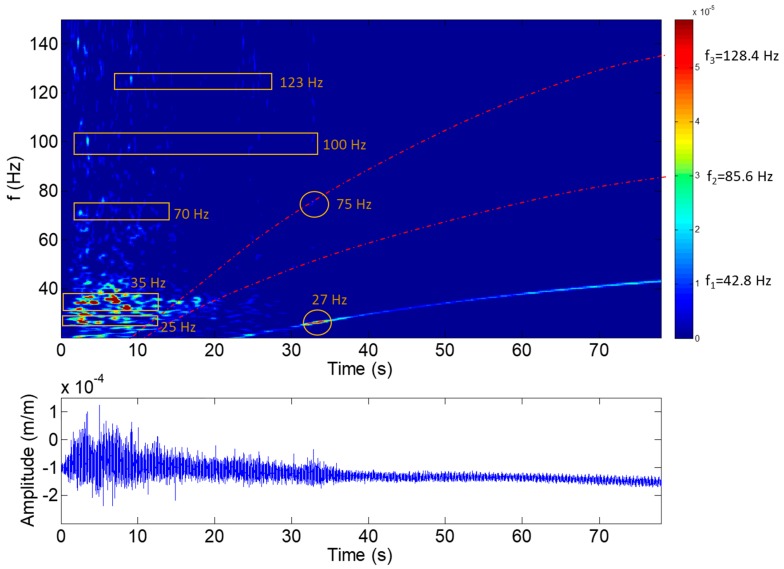
Joint time–frequency plot of a strain-gauge signal (crown side, pressure side, blade 7) during the initial hit.

During the start-up, the natural frequencies may be excited first with the initial hit of the water to the runner and then with the RSI during the acceleration of the machine (see Figure 5). Figure 9 shows the results obtained for the joint time–frequency analysis using wavelets of a strain gauge located in the crown side and the pressure side of blade 7. It can be seen that many frequencies are excited during the first 10 s of the start-up, corresponding to the initial hit of the water on the runner. Four different frequencies can be identified at this moment: 25, 35, 70, 100 and 123 Hz. These frequencies may be the first natural frequencies of the runner in water and without rotation (at this moment, the runner is just starting to rotate). After the first 10 s of the start-up, the turbine starts to accelerate and the first excitation line of the RSI is clearly identified (f_1_). The second and third excitation lines due to the RSI are highlighted using a dotted red line (see Figure 9). During this acceleration, a frequency of 27 Hz is excited with high amplitude with the first excitation line (marked with a circle in the figure) and another frequency of 75 Hz is also excited with the third excitation line (again marked with a circle in the figure). These two frequencies seem to correspond, respectively, to those found at 25 Hz and 70 Hz in the beginning of the transient. The slight increase of these natural frequencies could be due to different amounts of cavitation between the initial hit and the acceleration of the machine, or because the natural frequencies of the runner are also affected by the rotating speed of the machine, as Presas et al. found in their experimental study [12].

To confirm that these excited frequencies are natural frequencies of the runner, other operating conditions have been analyzed, as well as detection with the other strain gauges installed.

#### 5.2.2. Deep Part Load

**Figure 10:**
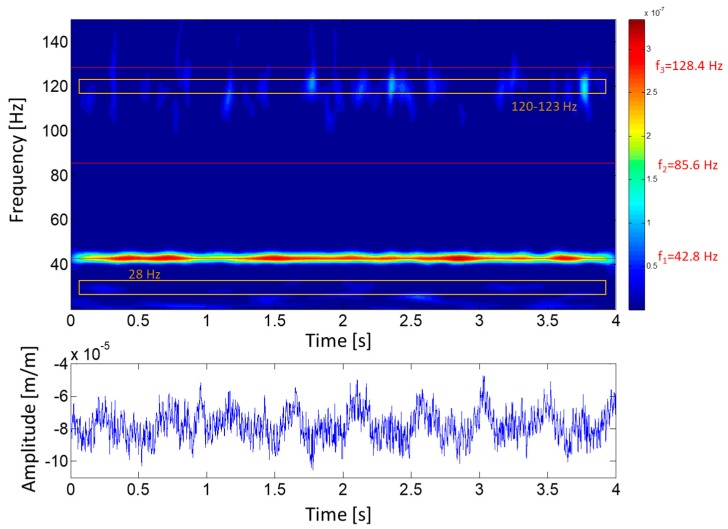
Joint time–frequency plot of a strain gauge signal (crown side, pressure side, blade 7) during DPL (18% of rated power).

During DPL, where the turbine operates with very low flow and power, the turbulence is one of the most important excitation phenomena (see Section 2.2.3). In this situation, the runner is excited randomly, hence some of its natural frequencies may be detected. Figure 10 shows the joint time-frequency (using wavelets) plot of 4 s of a strain gauge signal during DPL operation. A random excitation of some frequencies in the range of 120–123 Hz and with less amplitude in the zone of 28 Hz can be observed. These frequencies could be natural frequencies of the runner.

**Figure 11:**
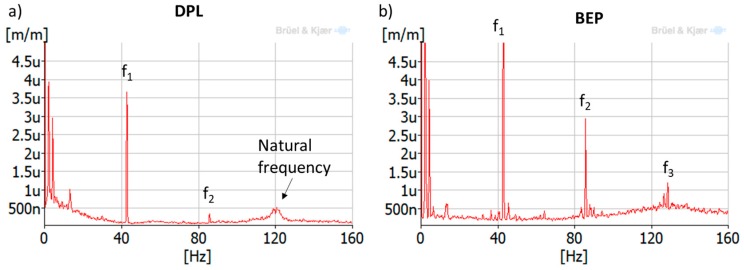
Averaged-spectrum analysis of a strain-gauge signal (crown side, pressure side, blade 7) during (**a**) DPL (18% of rated power); and (**b**) best efficiency point (BEP) (90% of rated power).

**Figure 12:**
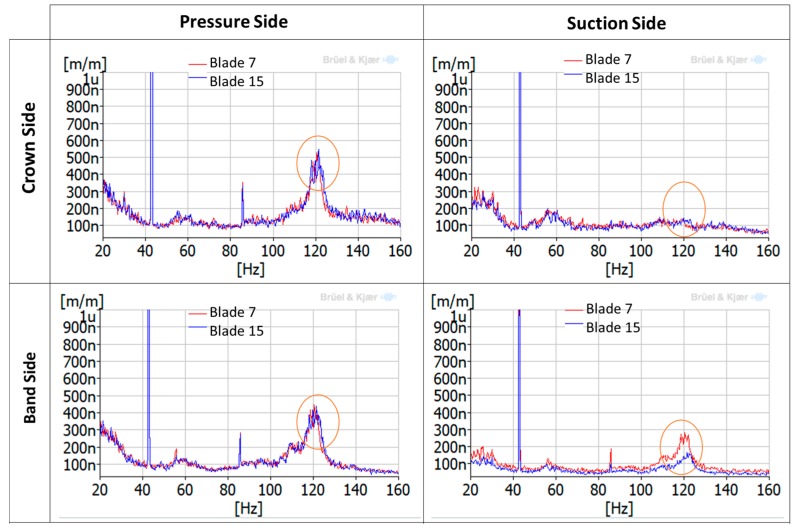
Averaged-spectrum analysis of a different strain gauges during DPL (18% of rated power).

Further analysis using the averaged-spectrum method were also applied to a larger time signal of the same strain gauge (see Figure 11). In this figure, the average of 100 spectra during 170 s was plotted for DPL operation and BEP operation. It can be seen that a zone between 120–123 Hz is clearly excited in DPL, but not in BEP. Moreover, looking at the different strain gauges installed in the runner (Figure 12), it can be clearly observed that this zone (120–123 Hz) is more excited in the pressure side than in the suction side (in the suction side it is only detected in the band strain gauges). This is the same behavior as obtained for the natural frequencies of the runner in air (Figure 8), so it can be confirmed that it is a natural frequency of the runner in water. Moreover, this natural frequency is rather near the third harmonic excitation of the RSI (f_3_ = 128.4 Hz), which according to Table 1 (column 3) is mainly a k_n.m_ = 4 excitation; so it seems that it is more excited than other frequencies because it may be a 4ND mode shape. According to the natural frequencies in air and previous knowledge about natural frequencies in water of Francis turbines [10,22,23,24], this natural frequency (120–123 Hz) may correspond to the mode shape called “4ND-CD-IPh” in Table 2. This means that this mode shape has a reduction of 52% of the natural frequency in air when in operating conditions.

#### 5.2.3. Overload

**Figure 13:**
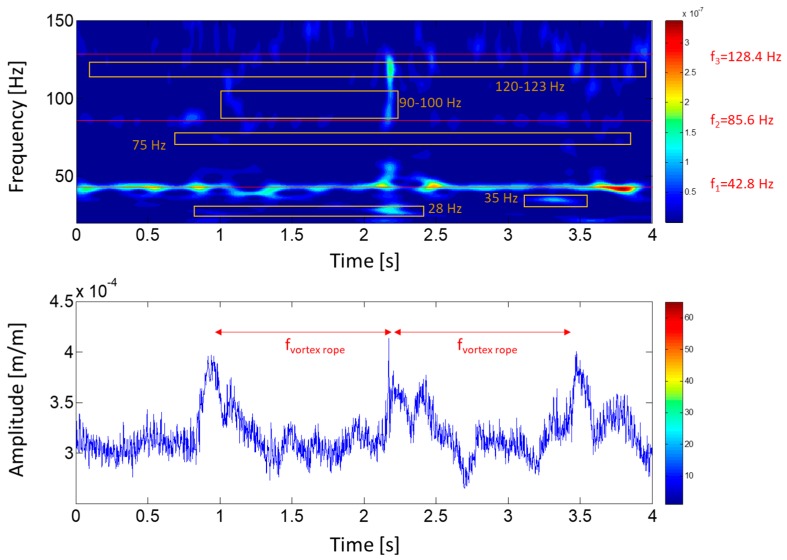
Joint time–frequency plot of a strain-gauge signal (crown side, pressure side, blade 7) during overload (OL) condition (107% of rated power).

During the OL operation, the runner receives large pressure pulses due to the overload vortex rope, which can act as strong impacts over the structure exciting all its natural frequencies (see Section 2.2.1). Figure 13 shows a joint time–frequency analysis of a strain-gauge signal during 4 s. The strong periodic (f_vortex rope_) fluctuations due to the vortex rope can be observed clearly in the time plot. Every maximum of these fluctuations correspond to a strong shake of the runner, exciting all its natural frequencies. Different frequencies are excited during this moment: 28 Hz, 35 Hz, 75 Hz, 90–100 Hz and 120–123 Hz.

All the frequencies excited under the three different operating conditions analyzed are listed in Table 3. Some of them can be correlated to a mode shape by means of analyzing phases and amplitudes between all gauges or excitation patterns. However, there are two that can correspond to different mode shapes, so they have not been classified. The ratio between the natural frequency in water and in air is also shown in Table 3. The values are of about 50–60% the natural frequency in air, which matches the expected values according to literature [10,22,23,24].

## 6. Conclusions

The feasibility of detecting natural frequencies of hydraulic turbine runners using strain gauges has been studied. Strain gauges are sometimes used to measure the stresses of hydraulic turbine runners in operation during commissioning, but they were never used to detect the natural frequencies of the runner. In this study, this is undertaken in a large hydraulic turbine prototype (444 MW of rated power). For that purpose, several strain gauges were installed in the runner and their signals were measured under different operating conditions.

First of all, strain modal testing (SMT) on the runner in air was performed and the results were compared with an experimental modal analysis (EMA) carried out using accelerometers. The first 12 natural frequencies and mode shapes of the runner in air were determined. After that, three different operating conditions of the hydraulic turbine were analyzed: the start-up, the overload, and the deep part load conditions. Different signal analysis methods were applied to every operating condition in order to detect the runner’s natural frequencies.

During the start-up of the machine, the natural frequencies of the runner were excited by means of the initial hit of the water on the runner. At this moment, some natural frequencies could be identified by means of a joint time–frequency analysis. Moreover, during acceleration of the machine, some of those natural frequencies were also excited by the rotor-stator interaction.

Similar behavior occurs during the overload condition. In this condition, the runner receives large pressure pulses produced by a cavitating vortex rope. These strong pressure pulses act as impacts, so all the runner’s natural frequencies are excited. The same natural frequencies as in the start-up were detected during the overload condition when applying a joint time–frequency analysis.

During the deep part load operation, when the runner is subjected to random excitation due to flow turbulence, two of the frequencies detected during the start-up and overload conditions were also excited. One of them (123 Hz) is clearly detected with a spectrum-averaged analysis in the different strain gauges. Thanks to the information obtained with the strain modal testing in air, this natural frequency could be identified as a 4ND mode shape.

The natural frequencies detected under operating conditions have been associated with those detected in air by means of the relative amplitudes and phases between strain gauges and previous knowledge of Francis turbines’ modal behavior. Analysis of the three operating conditions was necessary in order to correlate every natural frequency with the type of mode shape. The reduction of the natural frequencies under operating conditions in comparison with the natural frequencies of the runner in air is about 50–60% depending on the mode shape, values that match well some results obtained in literature by numerical simulations.

## Figures and Tables

**Table 1:** Excitation shape (k_m,n_) table for a combination of Z_b_ = 16 and Z_v_ = 20 runner blades. The most important components are colored in red.

mZv/nZb	20 (f_1_ = 42.8 Hz)	40 (f_2_ = 85.6 Hz)	60 (f_3_ = 128.4 Hz)	80 (f_4_ = 171.2 Hz)
16	−4	−24	−44	−64
32	12	−8	−28	−48
48	28	8	−12	−32
64	44	24	4	−16
80	60	40	20	0

**Table 2:** First 12 mode shapes, natural frequencies and damping ratios of the runner in air. Results obtained by means of the EMA.

Mode-Shape Number	Mode-Shape Name	f_n_ [Hz]	Damping Ratio [%]
1	2ND-G-IPh	46.44	1.0272
2	3ND-G-IPh	98.24	0.5498
3	1ND-G-IPh	129.14	2.4669
4	4ND-G-CPh	148.08	0.3094
5	2ND-BlD-IPh	155.58	0.3469
6	5ND-BlD-CPh	181.03	0.2713
7	3ND-BlD-IPh	192.48	0.2394
8	6ND-BlD-CPh	197.89	0.2894
9	0ND-BlD-IPh	206.85	0.2490
10	1ND-BlD-CPh	209.38	0.2413
11	1ND-BlD-IPh	216.99	0.8927
12	4ND-CD-IPh	233.25	0.2550

**Table 3:** Natural frequencies of the runner detected while in operation.

Frequency Detected in Operation (Hz)	Start-Up	DPL	OL	Mode Shape	f_air_ (Hz)	f_water_/f_air_
28	Yes	Yes	Yes	2ND-G-IPh	46.44	0.6029
35	Yes	No	Yes	-	-	
75	Yes	No	Yes	4ND-G-CPh	148.08	0.5065
100	Yes	No	Yes	-	-	
123	Yes	Yes	Yes	4ND-CD-IPh	233.25	0.5273

## Supplementary Materials

The following videos are available online at http://www.mdpi.com/1424-8220/18/1/174/s1, Video S1: 2ND-G-IPh, Video S2: 3ND-G-IPh, Video S3: 1ND-G-IPh, Video S4: 4ND-G-IPh, Video S5: 2ND-BlD-IPh, Video S6: 5ND-BlD-CPh, Video S7: 3ND-BlD-IPh, Video S8: 6ND-BlD-CPh, Video S9: 0ND-BlD-IPh, Video S10: 1ND-BlD-CPh, Video S11: 1ND-BlD-IPh, Video S12: 4ND-CD-IPh.
